# Progress Toward the Elimination of Hepatitis B and Hepatitis C in the Country of Georgia, April 2015–April 2024

**DOI:** 10.15585/mmwr.mm7330a1

**Published:** 2024-08-01

**Authors:** Rania A. Tohme, Shaun Shadaker, Ekaterine Adamia, Irma Khonelidze, Ketevan Stvilia, Vladimer Getia, Maia Tsereteli, Maia Alkhazashvili, Akaki Abutidze, Maia Butsashvili, Maka Gogia, Nancy Glass, Sophia Surguladze, Irina Tskhomelidze Schumacher, Tamar Gabunia

**Affiliations:** ^1^Division of Viral Hepatitis, National Center for HIV, Viral Hepatitis, STD, and TB Prevention, CDC; ^2^National Center for Disease Control and Public Health of Georgia, Tbilisi, Georgia; ^3^Infectious Diseases, AIDS and Clinical Immunology Research Center, Tbilisi, Georgia; ^4^Health Research Union, Tbilisi, Georgia; ^5^Georgian Harm Reduction Network, Tbilisi, Georgia; ^6^Training Programs in Epidemiology and Public Health Interventions Network, Tbilisi, Georgia; ^7^Ministry of Internally Displaced Persons from the Occupied Territories, Health, Labour and Social Affairs of Georgia, Tbilisi, Georgia.

**Summary**

**What is already known about this topic?**

Hepatitis B and hepatitis C are leading causes of cirrhosis and liver cancer. In April 2015, the country of Georgia launched a hepatitis C elimination program to address its high prevalence of hepatitis C.

**What is added by this report?**

As of April 2024, 83% of persons with chronic hepatitis C have received a diagnosis, and 86% of those diagnosed have initiated treatment. Sustained hepatitis B vaccination coverage above 90% has substantially reduced prevalence of infection in children; however, prevalence in adults remains high.

**What are the implications for public health practice?**

Identifying persons with chronic hepatitis C who have never received a diagnosis and linking them to care, and scaling up hepatitis B screening and treatment, would accelerate progress toward hepatitis B and hepatitis C elimination by 2030.

## Abstract

Hepatitis B and hepatitis C are leading causes of cirrhosis and liver cancer and caused 1.3 million deaths worldwide in 2022. Hepatitis B is preventable with vaccination, and hepatitis C is curable with direct-acting antivirals. In 2015, in collaboration with CDC and other partners, Georgia, a country at the intersection of Europe and Asia, launched a hepatitis C elimination program to reduce the prevalence of chronic hepatitis C; at that time, the prevalence was 5.4%, more than five times the global average of 1.0%. In 2016, the World Health Assembly endorsed a goal for the elimination of viral hepatitis as a public health problem by 2030. In 2024, 89% of the Georgian adult population have received screening for hepatitis C, 83% of persons with current chronic HCV infection have received a diagnosis, and 86% of those with diagnosed hepatitis C have started treatment. During 2015–2023, vaccination coverage with the hepatitis B birth dose and with 3 doses of hepatitis B vaccine among infants exceeded 90% for most years. In 2021, the prevalence of hepatitis B surface antigen was 0.03% among children and adolescents aged 5–17 years and 2.7% among adults. Georgia has demonstrated substantial progress toward hepatitis B and hepatitis C elimination. Using lessons from the hepatitis C elimination program, scale-up of screening and treatment for hepatitis B among adults would prevent further viral hepatitis–associated morbidity and mortality in Georgia and would accelerate progress toward hepatitis B and hepatitis C elimination by 2030.

## Introduction

Worldwide, in 2022, an estimated 304 million persons had chronic hepatitis B virus (HBV) or hepatitis C virus (HCV) infection, two leading causes of cirrhosis and liver cancer, and an estimated 2.2 million new HBV and HCV infections and 1.3 million associated deaths occurred (*1*). In 2016, the World Health Assembly endorsed the goal of eliminating viral hepatitis as a public health problem by 2030, defined as a 90% reduction in incidence of newly reported chronic infections (95% for hepatitis B and 80% for hepatitis C), and a 65% decrease in mortality compared with 2015 estimates (*2*). Elimination of HBV and HCV infections are documented at the country level by achievement of impact (disease incidence and mortality) targets and programmatic (infant vaccination coverage, prevention, diagnosis, and treatment) targets (*3*). Achievement of impact targets is demonstrated by 1) hepatitis B surface antigen (HBsAg)* seroprevalence of ≤0.1% among children aged ≤5 years, 2) an annual incidence of new chronic HCV infections of five or fewer per 100,000 persons in the general population and two or fewer per 100 among persons who inject drugs (PWID), and 3) an annual hepatitis B and hepatitis C combined mortality of six or fewer per 100,000 persons. Achievement of programmatic targets is demonstrated by 1) timely^†^ hepatitis B vaccine (HepB) birth dose (HepB-BD) and 3 infant doses of HepB (HepB3) coverage of ≥90%, 2) receipt of a diagnosis by ≥90% of persons with chronic hepatitis B and hepatitis C, 3) treatment of ≥80% of persons with diagnosed hepatitis B and hepatitis C, 4) 100% safe injections,^§^ 5) 100% blood safety,^¶^ and 6) distribution of 300 needles and syringes per PWID per year** (*3*).

Georgia, a country in the Caucasus region, had a prevalence of chronic HCV infection of 5.4% in 2015, compared with a global average of 1.0% (*4*). In April 2015, in collaboration with CDC and other partners, Georgia launched the world’s first hepatitis C elimination program. Modeled estimates using prevalence from nationwide serologic surveys conducted in 2015 and 2021 indicate that 130,000 Georgians were estimated to have current chronic HCV infection in 2015, and 77,000 were estimated to have chronic HBV infection in 2021 (*5*,*6*). In April 2024, Georgia’s government endorsed a hepatitis B elimination program. This report describes the progress made during April 2015–April 2024 toward achieving hepatitis B and hepatitis C elimination in Georgia.

## Methods

### Data Sources: Impact Indicators

Data on chronic hepatitis B and chronic hepatitis C seroprevalence were compiled from published nationwide serologic surveys (*6**,**7*). Incidence of new chronic HCV infections was based on modeled estimates derived from the 2021 nationwide serologic survey findings (*5*–*7*).

### Data Sources: Programmatic Indicators

Official country-reported^††^ timely HepB-BD and HepB3 vaccination coverage data were compiled. Data from the national hepatitis C screening and treatment registries were used to estimate the number of persons who received testing and treatment for hepatitis C. Population data were obtained from the national statistics office.^§§^ Vital statistics registry data were used to estimate combined hepatitis B and hepatitis C mortality. Because no national hepatitis B screening and treatment registry exists, the number of persons screened for hepatitis B was estimated using data from the national hepatitis C screening and treatment registries, because all patients with current chronic HCV infection should be screened for hepatitis B, and from persons screened at harm reduction sites.^¶¶^ Data on injection safety, blood safety, and indicators of services provided to PWID (e.g., the number of syringes and needles distributed per PWID and coverage with opioid agonist treatment) were obtained from relevant programs in Georgia.

### Analysis

The percentages of persons in Georgia who received a screening test for hepatitis C were calculated using the numbers from the national hepatitis C screening registry as numerators and national population statistics as denominators. A unique national identification number, common across hepatitis C screening and treatment registries, was used to track the progression of persons with hepatitis C in the care cascade. SAS software (version 9.4; SAS Institute) was used to analyze the hepatitis C care cascade. The number of syringes and needles distributed per PWID each year was calculated by dividing the total number of syringes and needles distributed to harm reduction sites by the total number of PWID registered at harm reduction sites. Coverage with opioid agonist treatment was calculated by dividing the number of PWID who received opioid agonist treatment by the estimated number of PWID in Georgia. This activity was reviewed by CDC, deemed not research, and was conducted consistent with applicable federal law and CDC policy.***

## Results

### Impact Indicators for Hepatitis B and Hepatitis C Elimination

The 2021 nationwide serologic survey found HBsAg seroprevalence of 0.03% (95% CI = 0%–0.19%) among children and adolescents aged 5–17 years and 2.7% (95% CI = 2.3%–3.4%) among adults. The same serologic survey reported a chronic hepatitis C prevalence of 1.8% (95% CI = 1.3%–2.4%) among adults, indicating a 67% decrease in prevalence compared with 5.4% in 2015. Modeled estimates based on the 2021 serologic survey results showed a decrease in the estimated annual incidence of new chronic HCV infections from 132 to 52 per 100,000 persons among the general non-PWID population and from 2.51 to 1.14 per 100 PWID during 2015–2022. Using national death registry data, researchers concluded that combined mortality from hepatitis B and hepatitis C increased from 6.3 per 100,000 persons in 2015 to 7.8 per 100,000 persons in 2023 (Table).

**TABLE:** Impact and programmatic indicators* for achievement of hepatitis B and hepatitis C elimination — Georgia, 2024

Indicator	Measure and target	Most recent data from Georgia
**Impact indicators**		
**Hepatitis B incidence (HBsAg seroprevalence)**	≤0.1% (persons aged ≤5 yrs)^†^	0.03% (95% CI = 0–0.19) (persons aged 5–17 yrs)^§^
**Hepatitis C incidence**		
Annual incidence of new chronic HCV infections per 100,000 general population	≤5	52^¶^
Annual incidence of new chronic HCV infections per 100 PWID	≤2	1.14^¶^
**Annual crude HBV and HCV mortality (deaths per 100,000 population)**	≤6	7.85
**Programmatic indicators**		
**Testing**		
Persons with chronic HCV infection who received a diagnosis, %	≥90	82.77
Persons with chronic HBV infection who received a diagnosis, %	≥90	3.46
**Treatment**		
Persons with diagnosed HCV infection who have initiated treatment, %	≥80	86.06
Persons with diagnosed HBV infection and eligible for treatment who are treated, %	≥80	NA
**Prevention**		
Timely** HepB-BD coverage, %	≥90	93^††^
Coverage with HepB3, %	≥90	92^††^
Safe injections in health care settings, %^§§^	100	100^¶¶^
Blood units screened for hepatitis B and hepatitis C, %	100	100
No. of syringes and needles distributed per PWID per year or OAT coverage among PWID***	≥300 per PWID per year or OAT coverage ≥40%	131 per PWID per year OAT coverage = 31.93%

**Abbreviations:** HBsAg = hepatitis B surface antigen; HBV = hepatitis B virus; HCV = hepatitis C virus; HepB3 = 3 infant doses of hepatitis B vaccine; HepB-BD = hepatitis B vaccine, birth dose; NA = not available; OAT = opioid agonist treatment; PWID = persons who inject drugs.

* https://iris.who.int/bitstream/handle/10665/373186/9789240078635-eng.pdf?sequence=1

^†^ HBsAg seroprevalence can be measured among children aged 1 year, 5 years, or 1–5 years, according to existing country surveillance and data collection practices. For regions and countries with a long history of high hepatitis B vaccination coverage and those that already conduct school-based serosurveys, serosurveys might be conducted in children aged >5 years. https://www.who.int/publications/i/item/9789240039360

^§^ https://www.eurosurveillance.org/content/10.2807/1560-7917.ES.2023.28.30.2200837

^¶^ https://www.eurosurveillance.org/content/10.2807/1560-7917.ES.2023.28.30.2200952

** Administration of a dose of hepatitis B vaccine within 24 hours of birth.

^††^ https://immunizationdata.who.int/global/wiise-detail-page/hepatitis-b-vaccination-coverage?CODE=GEO&ANTIGEN=HEPB3+HEPB_BD&YEAR=

^§§^ An alternate measure is ≥90% of syringes procured with an auto-disable function.

^¶¶^ CDC and ICAP Columbia University (https://icap.columbia.edu), Situational Analysis of Core Components of Infection Prevention and Control Programs at the Facility Level in Georgia, unpublished data, 2018.

*** An alternate measure in countries with opioid epidemics is OAT coverage among PWID ≥40%.

### Programmatic Indicators for Hepatitis B and Hepatitis C Elimination

**FIGURE 1:**
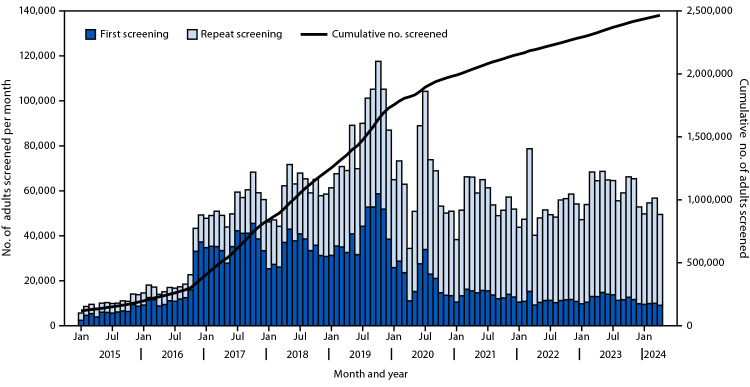
Monthly number of adults screened for hepatitis C virus antibody, by first and repeat screening status* — Georgia, January 2015–April 2024 * First screening was defined by receipt of testing for the first time. Repeat screening was defined by a second or subsequent screening of record.

**FIGURE 2:**
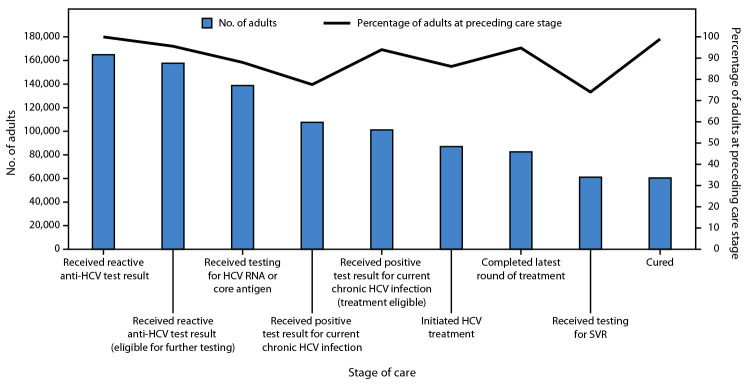
Hepatitis C cascade of care*^,^^†^^,^^§^^,^^¶^ among adults — Georgia, April 2015–April 2024 **Abbreviations**: anti-HCV = HCV antibody; HCV = hepatitis C virus; SVR = sustained virologic response. * Persons who received a reactive anti-HCV test result are among those with national identification numbers. An additional 18,586 persons using an anonymized 15-digit code had received a reactive anti-HCV test result. Thus, their representation in the cascade cannot be confirmed. ^†^ Eligible for further testing or treatment-eligible includes persons who received a reactive anti-HCV test result or who received a positive test result for current chronic HCV infection (before progressing in cascade) for whom no mortality data are available. ^§^ Approximately 130,000 persons are estimated to have current chronic HCV infection in Georgia. ^¶^ SVR is defined by the absence of detected HCV in blood ≥12 weeks after completing treatment.

**Diagnosis and treatment of current chronic HCV infection.** By April 2024, among the 2.8 million adults in Georgia, 2.5 million (89.3%) had been screened for HCV antibody (anti-HCV) (Figure 1). An additional 18,586 persons received a reactive anti-HCV test result using an anonymized code at harm reduction sites or prisons. Among adults screened for anti-HCV during January 2023–April 2024, 80% had been screened previously and consistently received nonreactive test results. Among the 2.5 million adults screened, 164,951 (6.7%) received a reactive anti-HCV test result. Among this group, 138,746 (84.1%) persons received testing for HCV presence (HCV RNA or HCV core antigen), of whom 107,604 (77.6%) had detectable HCV RNA or HCV core antigen, indicating current chronic infection. By April 2024, among 130,000 persons estimated to have current chronic HCV infection in Georgia, 107,604 (82.8%) had received a diagnosis of current chronic HCV infection. Among 101,138 (94.0%) of those 107,604 who were eligible^†††^ for treatment, 87,047 (86.1%) initiated treatment, 82,516 (94.8%) of whom completed treatment. Among 61,071 persons who received testing for sustained virologic response (absence of detected HCV RNA ≥12 weeks after completing treatment), 60,449 (99.0%) were cured of HCV infection (Figure 2). Overall, among 157,674 persons who received a reactive anti-HCV test result and were eligible for further testing, 18,928 (12.0%) did not receive an HCV RNA or HCV core antigen test. Among 101,138 persons who received a positive test result for current chronic HCV infection and were eligible for treatment, 14,091 (13.9%) did not initiate treatment.

**Diagnosis and treatment of chronic HBV infection.** Among 87,047 adults in Georgia treated for hepatitis C, 83,712 (96.2%) also received testing for HBsAg, among whom 1,978 (2.4%) received a positive test result. An additional 34,908 persons received HBsAg testing at harm reduction sites; 688 (2.0%) of these persons received a positive test result. Based on the estimated 77,000 persons in Georgia with chronic HBV infection, at least 2,666 (3.5%) had received a diagnosis by April 2024 (Table). While antiviral treatment (i.e., tenofovir disoproxil fumarate and entecavir) for hepatitis B is available in specialized clinics in Georgia, patients need to pay out of pocket for further assessment of treatment eligibility and to cover treatment costs. No hepatitis B screening and treatment registries exist to assess treatment coverage among eligible persons.

**Prevention of hepatitis B and hepatitis C.** Timely HepB-BD coverage remained consistently above 90% during 2015–2023. HepB3 coverage was above 90% for most years during 2015–2023, except during 2020–2022 when it dropped to 85%–88%. A 2018 independent assessment of infection prevention and control in 41 randomly selected hospitals across different regions in Georgia indicates that all facilities had continuous supply of single use and safety-engineered injection devices (CDC and ICAP Columbia University, Situational Analysis of Core Components of Infection Prevention and Control Programs at the Facility Level in Georgia, unpublished data, 2018). Since 2020, all blood units in Georgia have been screened for hepatitis B and hepatitis C by serology, and centralized nucleic acid testing is performed at the national reference laboratory. In addition, in 2022, Georgia adopted a law to strengthen the blood safety system to align with the European Union blood safety directives. In 2023, a total of 5,010,266 needles and syringes were distributed to 38,177 persons registered at harm reduction sites (131 needles and syringes per PWID) compared with a total of 3,611,785 needles and syringes distributed to 25,423 PWID in 2015 (142 needles and syringes per PWID) (Table). Among the estimated 49,700 PWID in Georgia,^§§§^ 15,869 (31.9%) received opioid agonist treatment through the national state program in 2023, a 167% increase compared with 5,953 (12%) in 2015.

## Discussion

Since launching its hepatitis C elimination program in April 2015, Georgia has made substantial progress in diagnosing and treating persons with HCV infection, improving the safety of the blood supply, and scaling up prevention services, leading to a 61% decrease in the estimated annual incidence of new chronic HCV infections (from 132 to 52 per 100,000 persons) among the general population and a 55% decline among PWID (from 2.51 to 1.14 per 100) (*5*). In recent years, more than 80% of adults received screening more than once for anti-HCV and received negative test results, suggesting that some persons who have current chronic HCV infection and need treatment might not have been reached yet. Hence, additional screening strategies, such as mobile screening campaigns, are needed to identify the remaining 17% of persons estimated to have current chronic HCV infection who have never received a diagnosis (*8*). In addition, many persons were lost to follow-up in the care cascade, highlighting the need to collect blood samples needed to test for anti-HCV and HCV RNA or HCV core antigen during the same visit. This practice will permit automatic HCV RNA or HCV core antigen testing among persons who receive a reactive anti-HCV test result, provision of adequate counseling so that clients can be informed of their test results, and referral of those who have current chronic HCV infection to care and treatment services (*9*). PWID and incarcerated populations have the highest prevalence of HCV infection in Georgia (*4*,*7*) and might be at risk for reinfection if prevention efforts, including increasing the number of needles and syringes distributed per PWID and access to opioid agonist treatment, are not further expanded.

Sustained high hepatitis B infant vaccination coverage for more than a decade has substantially reduced the incidence of HBV infection in children and adolescents. Current data demonstrate that Georgia might have achieved the targets for elimination of mother-to-child transmission of HBV (HBsAg seroprevalence of ≤0.1% among children and ≥90% HepB-BD and HepB3 coverage) (*3*). However, hepatitis B prevalence in adults remains high (*6*). To date, care and treatment for hepatitis B is not free, and there is no registry to document the care cascade. However, with the initiation of the hepatitis B elimination program, Georgia intends to provide free screening and treatment to all citizens starting in September 2024, and a national hepatitis B screening and treatment registry is under development.

Reported mortality from viral hepatitis has increased in Georgia; this is the result of improved reporting of causes of deaths in vital statistics registries and increasing hepatitis C screening, which has led to an increase in the number of persons who received a diagnosis of hepatitis C since 2015. However, further improvements in documenting causes of deaths resulting from viral hepatitis are needed to capture the combined mortality from hepatitis B and hepatitis C. This goal be achieved by implementing awareness and screening campaigns for hepatitis B and hepatitis C, and reporting mortality related to hepatitis B and hepatitis C in vital statistics registries.

### Limitations

The findings in this report are subject to at least four limitations. First, the total number of persons screened and linked to care for hepatitis C in the care cascade might be underestimated because data from harm reduction sites and prisons might not be fully incorporated in the national hepatitis C screening and treatment registries. Second, in the absence of a patient registry for hepatitis B, screening and treatment coverage might have been underestimated. Third, given that the majority (60%) of PWID in Georgia prefer buying needles and syringes from private pharmacies,^¶¶¶^ and that the data are lacking on procurement of needles and syringes outside of harm reduction sites, the number of needles and syringes provided per PWID was underestimated. Finally, combined mortality from hepatitis B and hepatitis C might be underestimated because of low screening for hepatitis B, limiting identification of HBV-related deaths.

### Implications for Public Health Practice

Identifying persons who have never been screened for hepatitis C, ensuring that those who receive a diagnosis are linked to care, and scaling up prevention services among PWID are essential to achieving HCV elimination in Georgia. Scaling up hepatitis B screening and treating those who are eligible for treatment would decrease hepatitis B–associated morbidity and mortality and expedite progress toward elimination by 2030. CDC is supporting and sharing lessons learned from Georgia’s efforts to eliminate hepatitis C with other countries in Eastern Europe and Central Asia that share similarly elevated prevalence of hepatitis C. 

## Footnotes

* HBsAg seropositivity is an indicator of chronic HBV infection. HBsAg seroprevalence can be measured among children aged 1 year, 5 years, or 1–5 years, according to existing country surveillance and data collection practices. Serosurveys might be conducted in children aged >5 years in regions and countries with a long history of high hepatitis B vaccination coverage and those that already conduct school-based serosurveys. https://www.who.int/publications/i/item/9789240039360

† Administration of a dose of hepatitis B vaccine within 24 hours of birth.

§ An alternate measure is ≥90% of syringes procured have an autodisable function.

¶ Donated blood units screened for bloodborne infections using quality-assured procedures.

** An alternate measure in countries with opioid epidemics is opioid agonist treatment coverage among PWID ≥40%.

††
https://immunizationdata.who.int/global/wiise-detail-page/hepatitis-b-vaccination-coverage?CODE%20=%20GEO&ANTIGEN%20=%20HEPB_BD+HEPB3&YEAR%20=

§§
https://www.geostat.ge/en/modules/categories/41/population

¶¶ An approach to policies, programs, or practices that is intended to reduce the negative health and social impacts of substance use.

*** 45 C.F.R. part 46, 21 C.F.R. part 56; 42 U.S.C. Sect. 241(d); 5 U.S.C. Sect. 552a; 44 U.S.C. Sect. 3501 et seq.

††† Not listed as deceased in any vital records.

§§§
https://www.inhsu.org/resource/population-size-estimation-of-people-who-inject-drugs-in-georgia/

¶¶¶
https://hri.global/wp-content/uploads/2023/11/LDSS-report_Final.pdf
